# Supplementary material for: Identification of Antimicrobial Peptides from the Microalgae Tetraselmis suecica (Kylin) Butcher and Bactericidal Activity Improvement
Source: Mar Drugs. 2019 Aug 1;17(8):453. doi: 10.3390/md17080453 (PMC6722934; doi:10.3390/md17080453)
Supplement: Supplementary file 1 [file marinedrugs-17-00453-s001.pdf]

Article

Identification of antimicrobial peptides from the microalgae *Tetraselmis suecica* (Kylin) Butcher, and bactericidal activity improvement.

Fanny Guzmán [1], Genezareth Wong [2], Tany Román [1], Constanza Cárdenas [1], Claudio Alvarez [3,4], Paulina Schimtt [4] , Fernando Albericio [5,6] and Verónica Rojas [2,*]

Supplementary Material

Table S1. Identity percentage of the sequences identified according BLAST search in NCBI, within Tetatraselmis genera (Tax id:).

#	Peptide	% Identity	NCBI accession number	Protein	Organism
**1**	**AQ-1749**	100	AMP43327	beta'' subunit of RNA polymerase (chloroplast)	*Tetraselmis sp.* CCMP 881
**2**	**AQ-1750**	100	CAA87754	NADH dehydrogenase subunit 5 (mitochondrion)	*Tetraselmis subcordiformis*
**3**	**AQ-1751**	--	--	--	--
**4**	**AQ-1752**	80	AML77346	putative LOV domain-containing protein	*Tetraselmis chui*
**5**	**AQ-1753**	80	ABA02341	photosystem II reaction center protein D1 (chloroplast)	*Tetraselmis suecica*
**6**	**AQ-1754**	83	AMP43289	apocytochrome f of cytochrome b6/f complex (chloroplast)	*Tetraselmis sp.* CCMP 881
**7**	**AQ-1755**	--	--	--	--
**8**	**AQ-1756**	80	AYF56515	heat shock protein 100	*Tetraselmis suecica*
**9**	**AQ-1757**	80	BAJ21550	beta-tubulin	*Tetraselmis chui*
**10**	**AQ-1758**	80	ABA02340	ATP synthase CF1 beta chain (chloroplast)	*Tetraselmis suecica*
**11**	**AQ-1759**	57	AML76832	putative LOV domain-containing protein	*Tetraselmis cordiformis*
**12**	**AQ-1760**	80	ABA02339	ribulose-1,5-bisphosphate carboxylase/oxygenase large subunit (chloroplast)	*Tetraselmis suecica*
**13**	**AQ-1761**	60	AFQ38466	ammonium transporter	*Tetraselmis chui*
**14**	**AQ-1762**	80	CAH59454	proton-translocating inorganic pyrophosphatase	*Tetraselmis suecica*
**15**	**AQ-1763**	60	AML77286	putative LOV domain-containing protein	*Tetraselmis striata*
**16**	**AQ-1764**	--	--	--	--
**17**	**AQ-1765**	60	AAO47330	high affinity phosphate transporter	*Tetraselmis chui*
**18**	**AQ-1766**	60	ABA02341	photosystem II reaction center protein D1	*Tetraselmis suecica*

**19**	**AQ-1767**	70	AMP43301	CP47 chlorophyll apoprotein of photosystem II (chloroplast)	*Tetraselmis sp.* CCMP 881
**20**	**AQ-1768**	--	--	--	--
**21**	**AQ-1769**	100	AFK27534.	cytochrome c oxidase subunit 2	*Tetraselmis gracilis*
**22**	**AQ-1770**	--	--	--	--
**23**	**AQ-1771**	--	--	--	--
**24**	**AQ-1772**	--	--	--	--

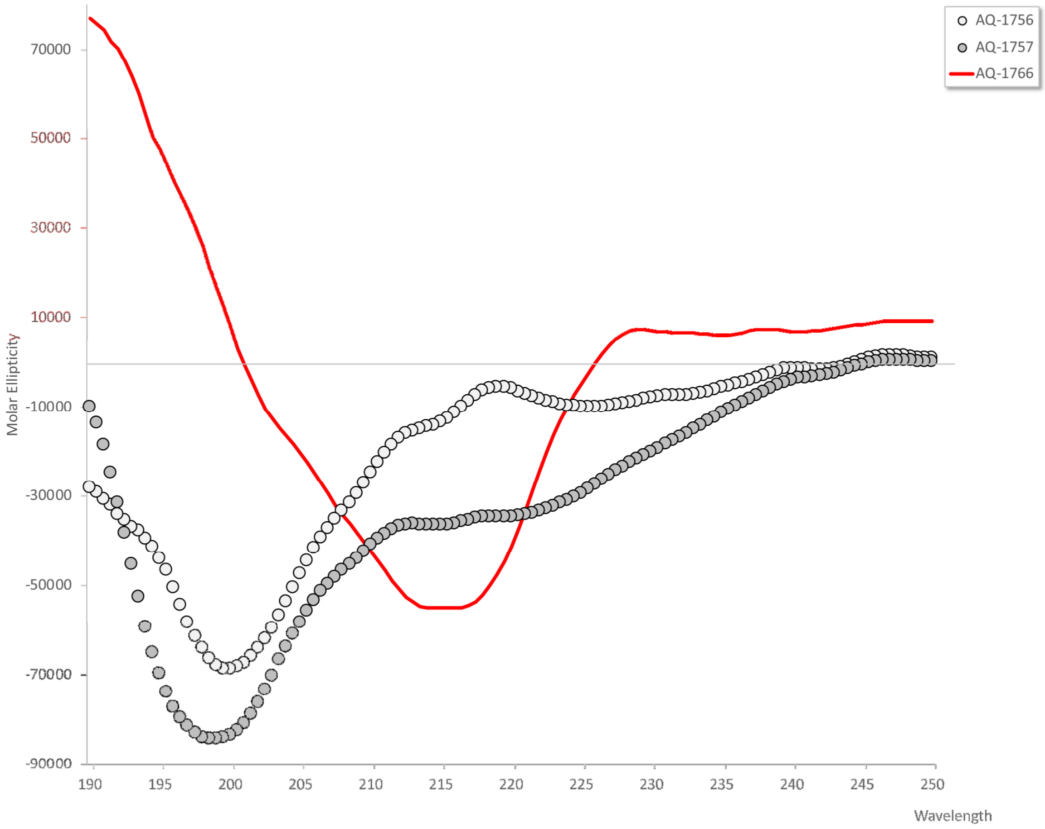

Figure S1. Circular dichroism of the peptides AQ-1755, AQ-1757 and AQ-1766. The spectra were performed in TFE 30%.

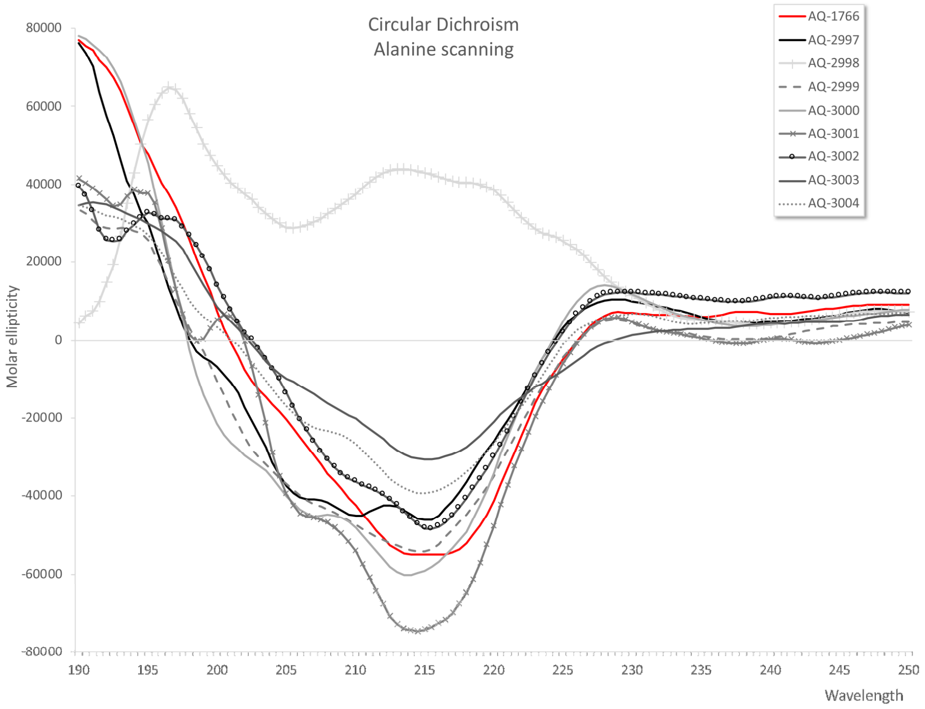

Figure S2. Circular dichroism of the peptide AQ-1766 (red line), and the analogous with alanine replacements (Table 2) . The spectra were performed in TFE 30%.

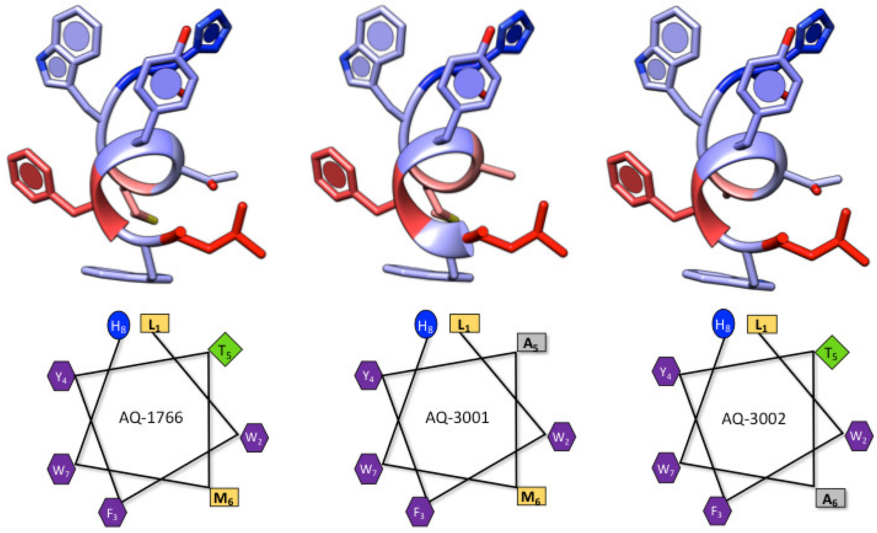

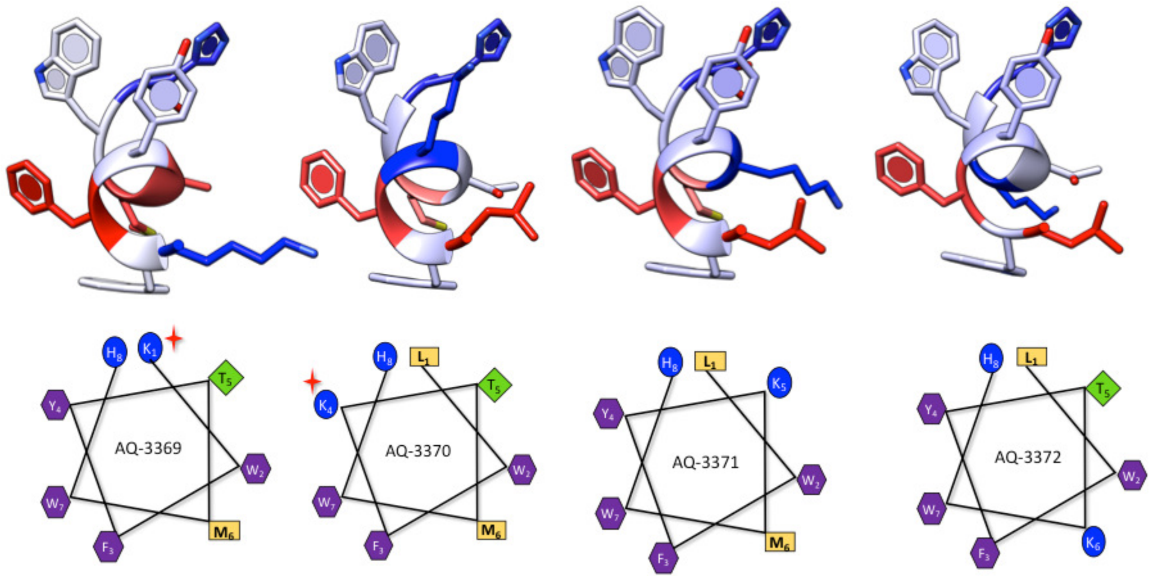

Figure S3. 3D and 2D representaton of active peptides: PEPFOLD3 prediction structures colored according to Kite & Doolitle hydrophobicity as implemented in Chimera, and helical wheel representations for each peptide modified from pepwheel of EMBOSS suite. Red stars indicate the lysine residues in the same face of the helix with the the other basic residue histidine.
